# Supplementary material for: Identification of Linear B Cell Epitopes on CD2V Protein of African Swine Fever Virus by Monoclonal Antibodies
Source: Microbiol Spectr. 2022 Mar 21;10(2):e01052-21. doi: 10.1128/spectrum.01052-21 (PMC9045250; doi:10.1128/spectrum.01052-21)
Supplement: SUPPLEMENTAL FILE 1 — Supplemental material. Download SPECTRUM01052-21_Supp_1_seq12.pdf, PDF file, 1.3 MB [file spectrum01052-21_supp_1_seq12.pdf]

MATERIALS AND METHODS

Bioinformatics analysis and modification of ASFV CD2V protein

According to the sequence of ASFV China/2018/ AnhuixCGQ isolates (Genebank No. MK128995.1), the sequence of ASFV CD2V (Protein ID:EP402R AYW34030.1) gene was analyzed by TMHMM Server V.2.0 online software (http://www.cbs.dtu.dk/services/TMHMM/) to identify the extracellular region. The extracellular sequence of CD2V protein was linked with itself by a flexible linker (GGGGSGGGGSGGGGS) to form CD2V recombination protein.

The codon optimization of CD2V recombinant protein

The sequence of ASFV CD2V recombinant protein was optimized codons by NovoPro online software (https://www.novopro.cn/tools/codon-optimization). Then modified gene of CD2V recombinant protein synthesized as pUC57-CD2V in Shanghai Sangon Bioengineering Co., Ltd. (Shanghai, China).

Construction of a recombinant vector pFastBac^{TM}1-CD2V and development of Bacmids

The gene of ASFV CD2V recombination protein was amplified by PCR using specific the upstream and downstream primers in Table S1.Then the target gene was cloned into the vector pFastBac^{TM}1 (Invitrogen, US) of the Bac-to-Bac baculovirus expression system (Invitrogen, US) by double digestion and ligation. The gp67 signal peptide was added to C terminus of the protein for secretory expression. Meanwhile, a 6×His tag was added to N terminus of the protein to facilitate further detection and purification. After double digestion, DNA ligation and transformation experiments, the PCR reaction was carried on using universal primers M13 (M13-For: 5'-CCCAGTCACGACGTTGTAAAACG-3', M13-Back: 5'-AGCGGATAACAATTTCACACAGG-3'). Two positive clones was selected and sequenced. Sequencing results were aligned with target gene using the Meglin software (DNAstar, US). Plasmids sequenced correctly are used to prepare Bacmids.

Analysis of immunogenicity for CD2V recombinant protein

Healthy female BALB/c mice, which were about 20g and 6-8 weeks old, were selected as immune experimental animals. The experimental group and the control group were set with five mice in each group. 5 μg/mouse CD2V recombinant protein was as immunogen in the experimental group, and the immunogen of the control group was sterilized PBS. Freund's complete adjuvant was used for the first immunization, and incomplete Freund's adjuvant was used for following two immunizations. Three immunizations were carried out in total. And the interval of immunization was 3 weeks (21 days). Serum was got by cutting tail at 63 days. The titer of antibody to CD2V recombinant protein in serum was detected using indirect ELISA method.

Amplifying variable regions of heavy chain and light chain of monoclonal antibodies

RNA of cells producing the five monoclonal antibodies was extracted with TRIzol (Invitrogen, Thermofisher,US) and reverse-transcribed into cDNA by PrimeScript™ RT Master Mix (Takara, Dalian, China). Nucleic acids of the heavy and light chains of CD2V mAbs were amplified by PCR using the primers in Table S2, and the results were observed by 2% agarose gel electrophoresis.

Prediction of CD2V B cell epitopes

Continuous B cell epitope regions of ASFV CD2V proteins were predicted using four bioinformatics softwares--ABCPred, BCPREDS, BepiPred and SVMTriP. Continuous B cell epitope regions of ASFV CD2V proteins were determined by comparison of the predicted results of each software.

RESULTS

Bioinformatics analysis and modification of ASFV CD2V protein

As was shown in Fig. S1 A and B, 1-206 amino acids (aa) was extracellular area including 1-16 aa CD2V signal peptide sequence. 207-229 aa was across the membrane area, and 230-360 aa was intracellular area. This result was consistent with the topology of ASFV CD2V in the Uniprot protein database (Fig. S1C). According to

ProtParam online prediction software, the isoelectric point (PI) of CD2V full-length sequence was 6.21 and the molecular weight was 41.008kDa, while the PI of CD2V extracellular region (1-206aa) was 4.65 and the molecular weight was 23.68kDa. CD2V recombinant protein was designed using a flexible linker (GGGGSGGGGSGGGGS) coupling with two homologous CD2V extracellular sequences in figure S1D. The PI of CD2V recombinant protein was 5.36 and the molecular weight was 45.62kDa by ProtParam tool of Expasy.

The codon optimization of CD2V-dimer sequence

The result of codon optimization for the original amino acid sequence of CD2V was shown in Figure S2. The codon optimization increased the CAI value to 0.94 (Fig. S2B) and the average GC content to 49.34% (Fig. S2C) in order that CD2V could be expressed well in eukaryotic baculovirus expression system.

Construction of a recombinant vector pFastBac™1-CD2V and development of Bacmids

As was shown in Figure S3A, 1% agarose gel electrophoresis showed that sequence of the CD2V recombinant protein was 1362 bp. As was shown in Figure S3B, two positive clones, pFastBac™1-CD2V-1 and pFastBac™1-CD2V-6, were located on correct size (3662 bp). The sequences of pFastBac™1-CD2V-1 and pFastBac™1-CD2V-6 were consistent with the CD2V target gene sequence. PCR results of 14 clones from blue-white selection were all positive clones. pFastBac™1-CD2V(DH10Bac)-1 was selected to prepare Bacmid.

Immunogenicity analysis of ASFV CD2V recombinant protein

As was shown in Figure S4, the antibody titer of CD2V recombinant protein as immunogen was 1:12,800 after third immunization. The OD450 values of negative control were lower than 0.5.

Amplification of variable region of heavy and light chain in monoclonal antibodies

As was shown in Figure S5, the sizes of the heavy and light chain were approximately 300 bp. And the heavy chain was slightly larger than the light chain. The CDR regions of mAbs were verified by IMGT. These analyses of IMGT were shown in Figure S6.

Bioinformatics prediction of ASFV CD2V protein B cell epitopes

As was shown in Figure S7A, ABCPred predicted continuous B cell epitopes on the extracellular region of the CD2V protein as the score from high to low: ^{152}NESILEYNWNNSNINN^{166},^{69}YSTSIYNITNNCSLTI^{84},^{57}CGKAGNFCECSNYSTS^{72},^{25}TIILDSNITNDNNDIN^{40},^{13}VLSIDYWVSFNKTIIL^{16},^{175}NNTISTSNETTLINCT^{190},^{100}NQIINYTIKLLTPATP^{115},^{38}DINGVSWNFFNNSFNT^{53},^{79}NCSLTIFPHNDVFDTT^{94},^{109}LLTPATPPNITYNCTN^{124},^{138}NIYLNINDTFVKYTNE^{153},^{115}PPNITYNCTNFLITCK^{130},^{123}TNFLITCKKNNGTNTN^{138},^{90}VFDTTYQVVWNQIINY^{105},^{162}NSNINNFTATCIINNT^{177},^{129}CKKNNGTNTNIYLNIN^{134},^{185}TLINCTYLTLSSNYFY^{200},^{31}NITNDNNDINGVSWNF^{46}.

As was shown in Figure S7B, continuous B cell epitopes on the extracellular region of the CD2V protein were predicted by BCPred Predictions software. B cell epitopes were shown as the scores from high to low: ^{171}TCIINNTISTSNETTLINCT^{190}, score: 0.999, ^{110}LTPATPPNITYNCTNFLITC^{129}, score: 0.996, ^{22}FNKTIILDSNITNDNNDING^{42}, score: 0.985, ^{150}YTNESILEYNWNNSNINNFT^{169}, score: 0.958, ^{43}SWNFFNNSFNTLATCGKAGN^{62}, score: 0.824, ^{65}ECSNYSTSIYNITNNCSLTI^{84}, score: 0.819.

The results of BepiPred software prediction were shown in Figure S7C. And B cell epitopes on the extracellular region of the CD2V protein were as follows: ^{12}LDSNITNDNNDINGVSWNFFNNSF^{35},^{40}TCGKAGNFCECSNYSTSIYNI^{60},^{71}HNDVFDTTYQV^{81},^{116}NNGTN^{120},^{131}FVKYTNE^{137},^{145}NNSNINNF^{152},^{176}LTLSSN^{181}.

As was shown in Figure S6D, the B cell epitopes predicted by the SVMTriP softwareas were as follows:

^{96}QVVWNQIINYTIKLLTPATP^{115}.

The results predicted by four softwares was compared with each other to find that ^{145}NNSNINNF^{152} was predicted as B cell epitope in three softwares. Hence, ^{145}NNSNINNF^{152} was the most possible B cells epitope on CD2V protein.

TABLE

Table S1 Primers for amplifying target genes of CD2V recombinant protein

Primers	Sequences（5’-3’）
CD2V-for	*GGATCC*ATGAAGTTCCTGGTTAACGTGGCT
CD2V-back	*TCTAGA*TTAGTGGTGGTGGTGATGGTGGTACAGCTTGAAGAAGGTGTA

Table S2 Sequences of primers to amplifying the variable region of heavy and light chains of monoclonal antibodies

Primers	Sequence
VH-For	5’-TGA GGA GAC GGT GAC CGT GGT CCC TTG GCC CC-3’
VH-Back	5’-AGG TSM ARC TGC AGS AGT CWGG-3’
VLFor1	5’-CCG TTT GAT TTC CAG CTT GGT GCC-3’
VLFor2	5’-CCG TTT TAT TTC CAG CTT GGT CCC-3’
VLFor3	5’-CCG TTT TAT TTC CAA CTT TGT CCC-3’
VLFor4	5’-CCG TTT CAG CTC CAG CTT GGT CCC-3’
VLBack	5’-GAC ATT GAG CTC ACC CAG TCT CCA-3’

FIGURE AND LEGENDS

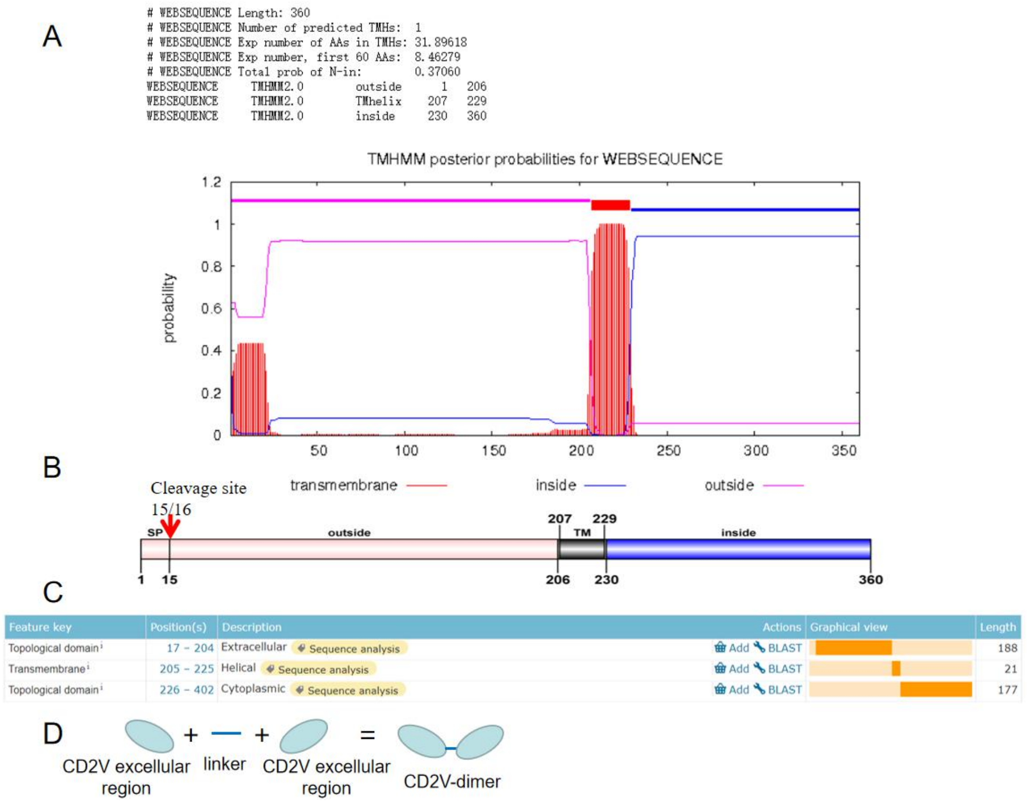

Figure S1. Prediction and modification of ASFV CD2V protein. (A and B) Transmembrane region of ASFV CD2V protein was predicted by TMHMM server V.2.0. 1-206 aa was extracellular area including 1-16 aa CD2V signal peptide sequence. 207-229 aa was across the membrane area, and 230-360 aa was intracellular area. (C) The topology of ASFV CD2V in the Uniprot protein database. (D) Two homologous CD2V extracellular regions were linked by a flexible linker (GGGGSGGGGSGGGGS) to form CD2V recombination protein.

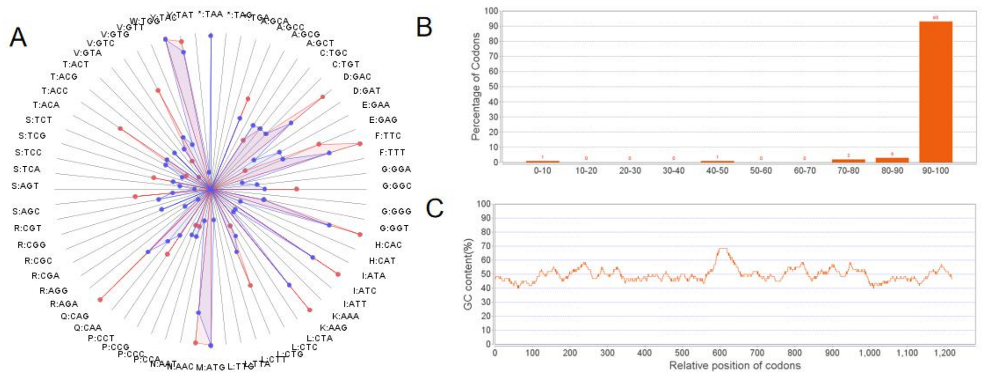

Figure S2 Codon optimization of CD2V recombinant protein in baculovirus expression system. (A) Codon relative frequency radar plot. (B) CAI value was increased to 0.94. (C) the average GC content was 49.34%.

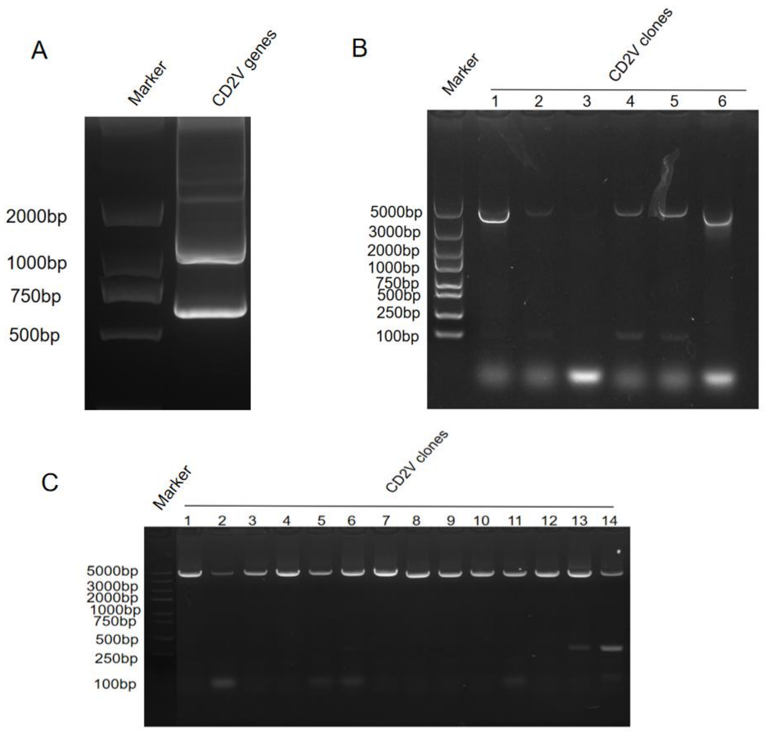

Figure S3. Construction of a recombinant vector pFastBac^{TM}1-CD2V and development of Bacmid. (A) Target genes of ASFV CD2V recombinant protein was amplified by PCR. (B) PCR results of recombinant plasmid pFastBac^{TM}1-CD2V. (C) PCR results of the white monoclonal bacteria screened by blue and white spots for Bacmids.

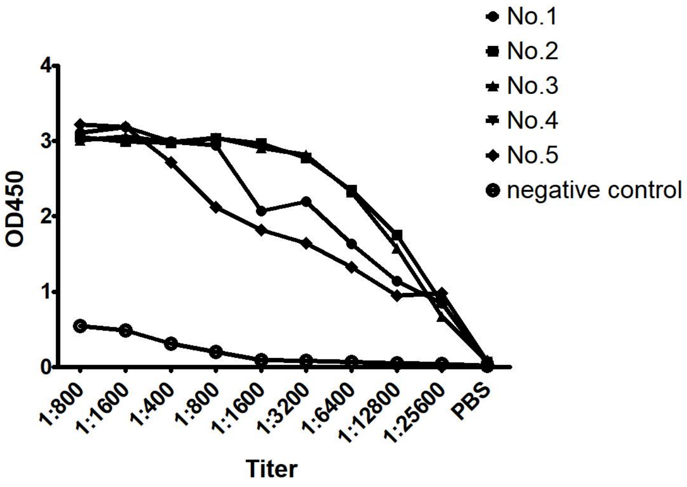

Figure S4. The antibody titer of CD2V recombinant protein as immunogen in serum sampled after third immunization. The immunogenicity titer of CD2V recombinant protein was 1:12,800.

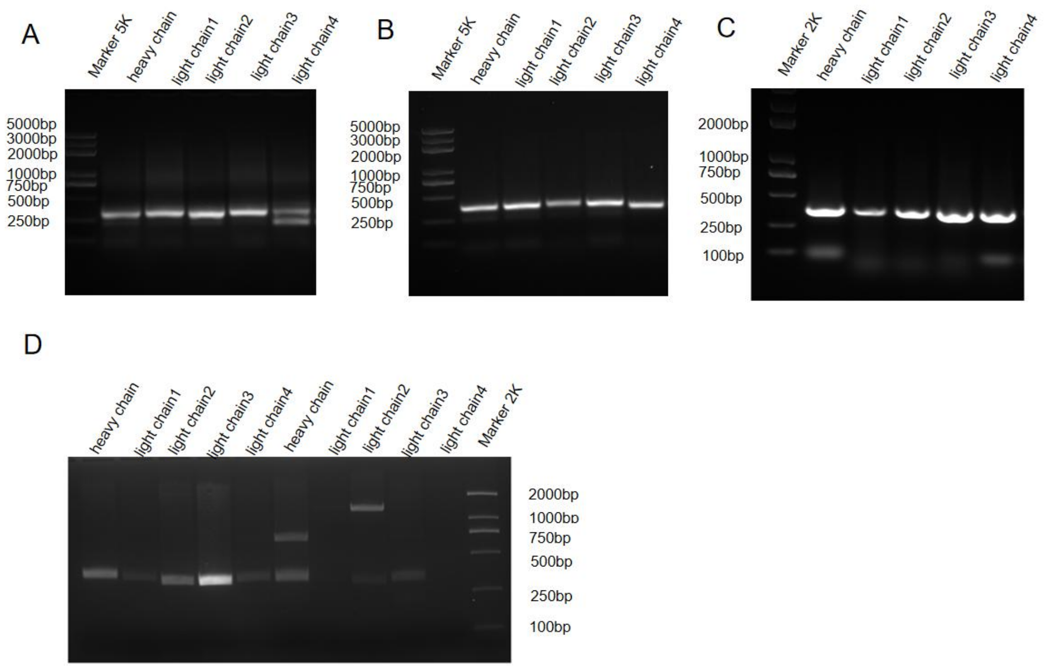

Figure S5 Amplification of variable region of heavy and light chain in monoclonal antibodies. Heavy and Light chains of five mAbs separated by 2% agarose gel electrophoresis after PCR amplification. (A-D) were 7E12 (A), 22B3 (B), 13G11 (C), 18A3 (D line 1-5), and 43C2 (D line 6-10), respectively.

A

```
FR1-IMGT     CDR1-IMGT   FR2-IMGT     CDR2-IMGT    FR3-IMGT      CDR3-IMGT   FR4-IMGT
(1-26)       (27-38)     (39-55)      (56-65)      (66-104)      (105-117)   (118-128)
A        B        BC        C        C'        C'C''     C''      D        E        F         FG         G
(1-15)   (16-26)  (27-38)   (39-46)  (47-55)   (56-65)   (66-74)  (75-84)  (85-96)  (97-104)  (105-117)  (118-128)
1  10 15 16  23 26 27  38 3941 46 47  55 56  65 66  74 75  84 85 89  96 97  104 105 11112 117 118  128
18A3  ..........MVKP GASVKISCKAS GYSF....TSYY IHWVKQRP GQGLEWIGW IFPG..SGNT KYNEKFK.G KATLTADTSS STAYMQLSSLTS EDSAVYFC AQTGR....VFAY WGQGTTVTVSS
```

B

```
1  10 15 16  23 26 27  38 3941 46 47  55 56  65 66  74 75  84 85 89  96 97  104 105 11112 117 118  128
18A3  ...MTQSPSSLSASL GERVSLTCRAS QEI......SGY LSWLQQKP DGTIKRLIY AA.......S TLDSGVP.K RFSGSR..SG SDYSLTISSLES EDFADYYC LQYAS....YPWT FGGGTKLE...
```

C

```
1  10 15 16  23 26 27  38 3941 46 47  55 56  65 66  74 75  84 85 89  96 97  104 105 11112 117 118  128
22B3  .VQLQESGG.GLVKP GGSLKLSCAAS GFTF....SSYA MSWVRQTP EKRLEWVAS ISSG...GST YYPDSVK.G RFTISRDNAR NILYLQMSSLRS EDTAMYFC ARRYRY.DAWFAY WGQGTTVTVSS
```

D

```
1  10 15 16  23 26 27  38 3941 46 47  55 56  65 66  74 75  84 85 89  96 97  104 105 11112 117 118  128
22B3  ...MTQSPAILSASP GEKVTMTCRAS SSV.......SY MHWYQQKP GSSPKPWIY AT.......S NLASGVP.A RFSGSG..SG TSYSLTISRVEA EDVATYYC QQWSSNP.RGRSV EAPSWKSNG..
```

E

```
1  10 15 16  23 26 27  38 3941 46 47  55 56  65 66  74 75  84 85 89  96 97  104 105 111 |1221| 112 117 118  128
43C2  ....QQSGA.ELMKP GASVKISCKAT GYTF....STYW IEWVKQRP GHGLEWIGE ILPG..GGST NYNEKFK.G KAMFTADTSS NTAYMQLSSLAS EDSAVYYC ARVRYGNYGGNYYAMDY WGQGTTVTVSS
```

F

```
1  10 15 16  23 26 27  38 3941 46 47  55 56  65 66  74 75  84 85 89  96 97  104 105 11112 117 118  128
43C2  DIELTQSPASLAVSL GQRATISYRAS KSVST..SGYSY MHWNQQKP GQPPRLLIY LV.......S NLESGVP.A RFSGSG..SG TDFTLNIHPVEE EDAATYYC QHIREL.TRSE.. ...........
```

G

```
1  10 15 16  23 26 27  38 3941 46 47  55 56  65 66  74 75  84 85 89  96 97  104 105 111 |11| 112 117 118  128
13G11  .VQSQESGP.DLVKP SQSLSLTCTVT GYSIT...SGYS WHWIRQFP GNKLEWMGY IHYS...GVT NYNPSLK.S RISITRDTSK NQFFLQLNSVTT EDTATYYC ARAPLYYGNYVWFSY WGQGTTVTVSS
```

H

```
1  10 15 16  23 26 27  38 3941 46 47  55 56  65 66  74 75  84 85 89  96 97  104 105 11112 117 118  128
13G11  DIELTQSPASLSASV GETVTITCRAS GNI......HNY LAWYQQKQ GKSPQLLVY NA.......K TLADGVP.S RFSGSG..SG SQYSLKINSLQP EDFGSYYC QHFWS....TPWT FGGGTKLE...
```

I

```
1  10 15 16  23 26 27  38 3941 46 47  55 56  65 66  74 75  84 85 89  96 97  104 105 11112 117 118  128
7E12  EVQLQQSGA.ELVRP GASVKLSCKTS GYIF....TSYW IHWVKQRS GQGLEWIAR IYPG..TGST YYNEKFK.G KATLTADKSS STAYMQLSSLKS EDSAVYFC ARGKYGNLYYFDY WGQGTTVTVSS
```

J

```
1  10 15 16  23 26 27  38 3941 46 47  55 56  65 66  74 75  84 85 89  96 97  104 105 11112 117 118  128
7E12  DIELTQSPASLAVSL GQRATISYRAS KSVST..SGYSY MHWNQQKP GQPPRLLIY LV.......S NLESGVP.A RFSGSG..SG TDFTLNIHPVEE EDAATYYC QHIREL.TRSEGA ...........
```

Figure S6. CDR regions of mAbs verified by IMGT. A. CDR regions of heavy chain variable sequence on 18A3 mAbs. B. CDR regions of light chain variable sequence on 18A3 mAbs. C. CDR regions of heavy chain variable sequence on 22B3 mAbs. D. CDR regions of light chain variable sequence on 22B3 mAbs. E. CDR regions of heavy chain variable sequence on 43C2 mAbs. F. CDR regions of light chain variable sequence on 43C2 mAbs. G. CDR regions of heavy chain variable sequence on 13G11 mAbs. H. CDR regions of light chain variable sequence on 13G11 mAbs. I. CDR regions of heavy chain variable sequence on 7E12 mAbs. J. CDR regions of light chain variable sequence on 7E12 mAbs.

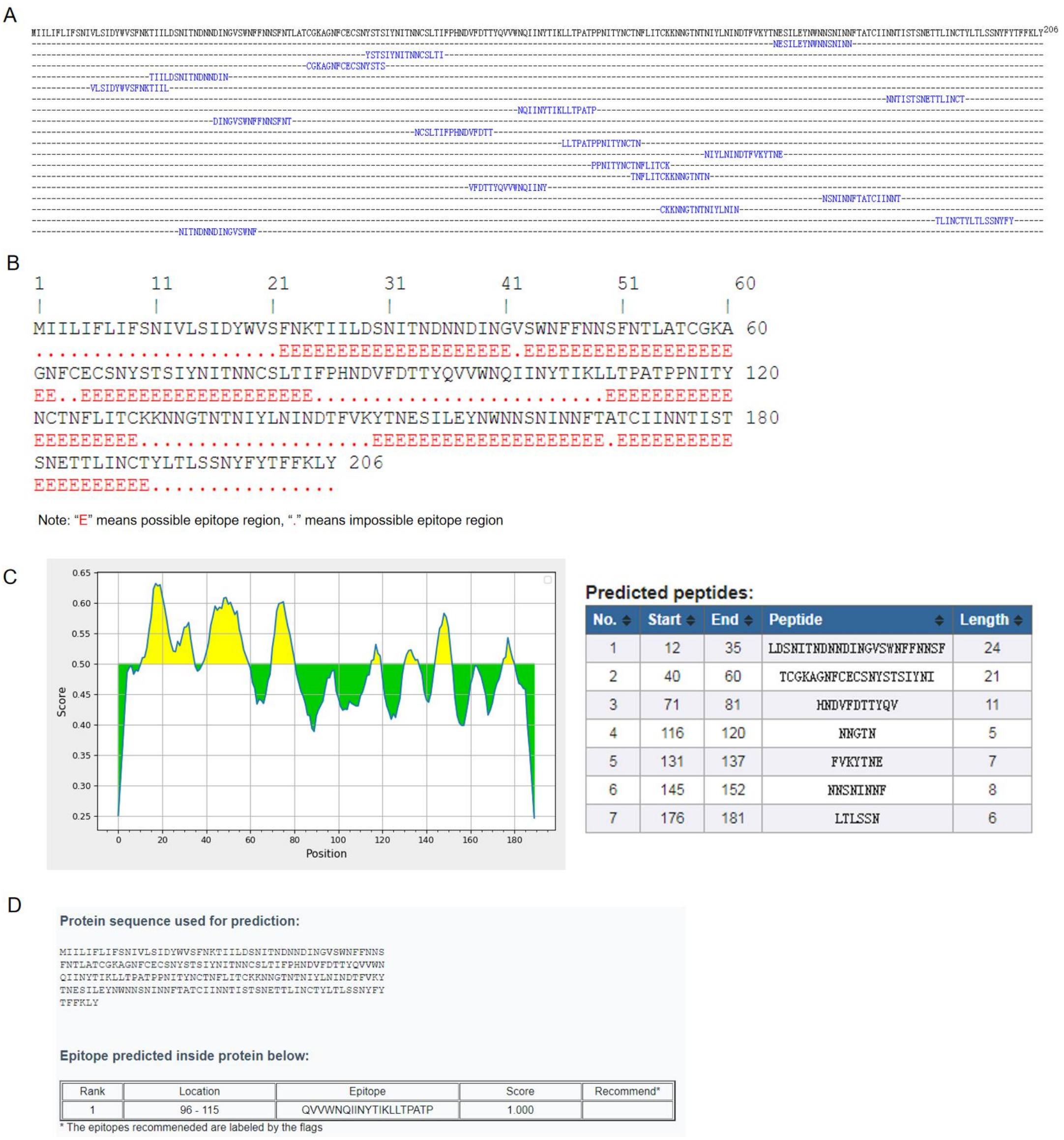

Figure S7. Bioinformatics prediction of ASFV CD2V protein B cell epitopes. (A) Continuous B cell epitopes of CD2V protein were predicted by ABCPred. (B) Continuous B-cell epitopes in the extracellular region of CD2V protein were predicted by BCPred Predictions software. (C) B cell epitope of ASFV CD2V protein were predicted by Bepipred software. The yellow regions were possible B cell epitope peptides. (D) SVMTriP software predicted the B cell epitopes of CD2V protein.
